# A qualitative investigation into the results of a discrete choice experiment and the impact of COVID-19 on patient preferences for virtual consultations

**DOI:** 10.1186/s40945-021-00115-0

**Published:** 2021-09-06

**Authors:** Anthony W. Gilbert, Carl R. May, Hazel Brown, Maria Stokes, Jeremy Jones

**Affiliations:** 1 Therapies Department, Royal National Orthopaedic Hospital, Stanmore, UK; 2 School of Health Sciences, Faculty of Environmental and Life Sciences, University of Southampton, Southampton, UK; 3 NIHR Applied Research Collaboration, North Thames, London, UK; 4 Faculty of Public Health and Policy, London School of Hygiene and Tropical Medicine, London, UK; 5 Centre for Nerve Engineering, UCL, London, UK; 6 NIHR Applied Research Collaboration, Wessex, UK; 7 Southampton NIHR Biomedical Research Centre, Southampton, UK

**Keywords:** Patient preferences, Virtual consultations, Telehealth, Orthopaedics, Rehabilitation, COVID-19

## Abstract

**Objectives:**

To conduct a qualitative investigation on a subset of participants from a previously completed Discrete Choice Experiment (DCE) to understand why factors identified from the DCE are important, how they influenced preference for virtual consultations (VC) and how COVID-19 has influenced preference for VC.

**Methods:**

A quota sample was recruited from participants who participated in our DCE. We specifically targeted participants who were strongly in favour of face-to-face consultations (F2F - defined as choosing all or mostly F2F in the DCE) or strongly in favour of virtual consultations (VC - defined as choosing all or mostly VC consultations in the DCE) to elicit a range of views. Interviews were conducted via telephone or videoconference, audio recorded, transcribed verbatim and uploaded into NVIVO software. A directed content analysis of transcripts was undertaken in accordance with a coding framework based on the results of the DCE and the impact of COVID-19 on preference.

**Results:**

Eight F2F and 5 VC participants were included. Shorter appointments were less ‘worth’ travelling in for than a longer appointment and rush hour travel had an effect on whether travelling was acceptable, particularly when patients experienced pain as a result of extended journeys. Socioeconomic factors such as cost of travel, paid time off work, access to equipment and support in its use was important. Physical examinations were preferable in the clinic whereas talking therapies were acceptable over VC. Several participants commented on how VC interferes with the patient-clinician relationship. VC during COVID-19 has provided patients with the opportunity to access their care virtually without the need for travel. For some, this was extremely positive.

**Conclusions:**

This study investigated the results of a previously completed DCE and the impact of COVID-19 on patient preferences for VC. Theoretically informative insights were gained to explain the results of the DCE. The use of VC during the COVID-19 pandemic provided opportunities to access care without the need for face-to-face social interactions. Many felt that VC would become more commonplace after the pandemic, whereas others were keen to return to F2F consultations as much as possible. This qualitative study provides additional context to the results of a previously completed DCE.

**Supplementary Information:**

The online version contains supplementary material available at 10.1186/s40945-021-00115-0.

## Key messages

What’s already known about this topic?

- The use of virtual consultations increased due to the COVID-19 pandemic.
- Several factors have previously been shown to influence patient preferences for virtual consultations.

What does the study add?

- This study provides theoretically informative insights to explain the results of a Discrete Choice Experiment.
- This study highlights the impact of COVID-19 on patient preferences for virtual consultations.

## Introduction

The UK’s National Health Service (NHS) Long Term Plan [1] sets out a policy agenda of mainstream digitally enabled care. Virtual Consultations (VC - either a real-time phone or a video consultation) have been suggested to reduce up to a third of outpatient appointments and save ‘over £1billion a year [1]. Benefits of VC also include saving patients time, convenience and freeing up healthcare professional time. The COVID-19 pandemic has accelerated the introduction of VC into clinical practice [2] with many organizations working hard to introduce VC [3]. Technology has taken a ‘central role’ [4] in healthcare following a ‘big bang’ change in technology driven work practices [5]. COVID-19 has brought about changes in the healthcare landscape in line with policy agenda in the NHS [1].

Virtual physiotherapy has seen an increase of interest following the pandemic. Prior to COVID-19, virtual physiotherapy was to have a number of advantages, including increased flexibility, accessibility and reduced costs [6]. Digitally enhanced outpatient care has been labelled as a positive legacy of COVID-19, with the use of VC opening the door to remote working, remote assessment, remote monitoring and rehabilitation [7]. Outpatient physiotherapy services are now seen to have the opportunity to use blended digital approaches with traditional face-to-face (F2F) appointments, to suit the needs of patients, in an individualized manner [8]. Although VC was embraced during the pandemic, less than half of clinicians in a cross-sectional survey believed telehealth was as effective as F2F care [9]. The development of effective, patient centered, accessible, equitable and flexible patient care pathways has been cited as an important ambition [10]. An understanding of patient preferences is essential to the design of such innovative pathways in physiotherapy.

Preferences can be defined as a ‘total subjective comparative evaluation’ [11]. Preferences are the result of a cognitive task whereby individuals consider the alternatives and their consequences to determine the option or action which yields the greatest utility (or benefit) to them. Rational preference theory assumes that the individual will subsequently choose the option which benefits them the most [11].

The CONNECT Project [12] is series of mixed methods studies investigating patient preferences for VC and is split across four phases. In Phase 1, a systematic review was conducted that investigated how the work of being a patient influences preferences for VC [13]. Phase 2 was a qualitative study that investigated the various factors that influence preferences for VC. Phase 3 extended this work through a discrete choice experiment (DCE) [14]; a deductive investigation to test the strength of individuals characteristics and demographic factors and their relationship with preference for VC. Our previous DCE was terminated prematurely due to COVID-19 and we are therefore treating the results as indicative rather than absolute as the required number of patients were not recruited to enable definitive conclusions to be drawn. The results of the DCE suggest that people who prefer VC are: more likely to have access to the equipment required to undertake a VC and to have difficulty with activities of daily living; less likely to have resources to accommodate time and travel and to be educated to degree level. Soon after the termination of the DCE, there was an organizational restructuring to introduce virtual consultations due to COVID-19 [2] and a qualitative investigation is needed to investigate the impact of COVID-19 on preferences and provide additional context to the results of the DCE [14].

The primary objective of the present study was to conduct a qualitative investigation on a subset of DCE respondents to understand why factors identified from the DCE are important, and how they influence preference for VC. A secondary objective was to understand how COVID-19 influences preference for virtual orthopaedic rehabilitation consultations.

## Methods

This research is a qualitative investigation to help us to further understand the results of phase III of the CONNECT project [14]. The CONNECT project protocol has previously been published [12].

### Ethics

Ethical approval for DCE delivery was obtained for Phase III (approval received on 18 October 2019 from the London-Hampstead Research Ethics Committee - IRAS ID: 248064, REC Reference 15/LO/1586). A subsequent amendment for inclusion of qualitative interviews was granted on the 26th June 2020. All participants provided informed written consent via email prior to inclusion.

### Setting

The research was conducted within a single specialist orthopaedic hospital in North London, UK. All participants were recruited from the Occupational Therapy and Physiotherapy Department.

### Participants

A quota sample was recruited from participants who completed our Discrete Choice Experiment (DCE) [14]. The inclusion criteria is demonstrated in Table 1.

**Table 1:** Inclusion and exclusion criteria

Inclusion criteria	Exclusion criteria
• Patients, over the age of 18 years, attending the hospital for Physiotherapy or Occupational Therapy^a^ • Patients who have experience of orthopaedic / musculoskeletal condition^a^ • Patients who are able to provide informed written consent • Patients able to understand and speak English or a language covered by the RNOH Interpreter service^a^ • Patients providing their contact details in Phase 3 [14] of the CONNECT project • Patients scoring 9/9 for F2F and at least 7/9 for VC	• Patients under the age of 18 years. • Patients without the capacity to consent^a^ • Patients suffering from diagnosis other than orthopaedic as the primary cause (eg neurological or oncology disorders)^a^ • Patients currently or previously treated by the lead investigator (AWG)^a^

^a^*denotes previous criteria used for the DCE*

### Recruitment

Participants meeting the inclusion criteria were sent an email by the lead investigator (AWG) informing them of the research. Those who replied indicating they were interested in taking part were sent the participant information sheet. Written consent to participate in the research was gained via email. A mutually convenient time was then arranged for interview.

### Data collection

Interviews were conducted via Zoom software or telephone. A topic guide, focusing on the results of the DCE, was used to facilitate discussions (see supplementary material 1). Interviews were audio recorded and transcribed verbatim.

### Data analysis

Transcripts were uploaded into QSR NVIVO (version 12). A directed content analysis [16] was undertaken in accordance with the coding framework designed from the results of Phase III [14] of the CONNECT project. This took the following form:

i. Data identified within the transcripts and allocated to the most appropriate factor group from the coding framework (pathway factors, clinical factors, socioeconomic factors, equipment factors, objective factors, interaction factors, COVID-19 impact on preference).
ii. Data were characterised based on the question: *how does this factor influence preference for virtual consultations?*
iii. The characterisation from (ii) was saved as a node within NVIVO.

Initial coding was undertaken by one author (AWG) with support from CRM. Another author (HB) subsequently reviewed all nodes within the NVIVO file to check that:

- Each node was an accurate representation of the interview transcript
- Each node fit within the coding framework.

Data were then presented with excerpts from transcripts to illustrate salient features.

### Coding frame

The coding frame is shown in Table 2. We were interested in data relating to:

i. How the context of the consultation (the circumstances of the consultation and the patient’s symptoms and activity levels) influences preference.
ii. How patient access to resources (based on their socioeconomic position and access to technological resources) influenced preference.
iii. How the requirements of the consultation (the objectives and whether the interactions required to fulfil the objectives) influence preference.
iv. The impact of COVID-19 on preference for F2F or VC.

**Table 2:** Coding Frame

	Context for consultation		Patient’s access to resources		Requirements of the consultation		How COVID-19 influences preference
	Pathway Factors	Clinical Factors	Socioeconomic Factors	Equipment Factors	Objective Factors	Interaction Factors	COVID-19 impact on preference
**Definition**	The circumstances of the consultation	The clinical context, including patient symptoms and activity levels.	The socioeconomic position of the patient.	The patients access to, and willingness to engage with, technology for a consultation	The requirements of the consultation.	Whether the patient feels the interactions required to fulfill the objectives of can be achieved with their clinician.	Whether COVID-19 changes the way patients feel about / prefer VC consultations
**Research Question**	How do pathway factors influence preferences for VC?	How do clinical factors influence preferences for VC?	How do socioeconomic factors influence preferences for VC?	How do equipment factors influence preferences for VC?	How do the requirements of the consultation influence preferences for VC?	How does the required clinical interaction influence preferences for VC?	How does the presence of COVID-19 influence preferences for VC?

## Results

### Respondents

Thirty-eight participants met the inclusion criteria from the F2F group. Of these, 26 did not respond, 4 declined interview and 8 were interviewed. Seventeen participants met the inclusion from the VC group. Of these 11 did not respond, 6 consented to interview with one participant subsequently unavailable for interview. Five were subsequently interviewed. Participant characteristics are demonstrated in Table 3. Interviews lasted for an average of 50 min (range 34 to 79 min). Empirical data are demonstrated in Table 4 (Context for the consultation), Table 5 (patient’s access to resources), Table 6 (what’s required from the consultation) and Table 7 (how COVID-19 influences preference).

**Table 3:** demographics of participants

	Prefer F2F	Prefer VC
Number	8	5
Gender	F = 5	F = 3
	M = 3	M = 2
Age	Average = 54 years; [range 38–79]	Average = 55 years; [range 20–75]
Ethnicity	White English = 5; Asian British = 1; Any other = 2; (Jewish =1, Mixed English = 1)	White English = 4; Asian British = 1
Highest Qualifications	School level qualifications = 4; Professional qualifications = 2; Apprenticeship = 1; Other = 1 (City & Guilds)	Degree (eg BSc, MSc) = 3; Professional qualifications = 2
Surgery for problem	No = 5; Yes = 3; (last month = 1; last three months = 1; last year = 1)	Yes = 5; (last month = 2; last year = 1; over a year ago = 2)
Condition restricting physical mobility	Yes = 5; No = 3	Yes = 5; No = 1
Symptoms	Upper limb = 2; Lower limb = 3; Spine / pelvis = 5	Upper limb = 3; Lower limb = 2; Spine / pelvis = 4
Access to VC equipment	Yes = 7	Yes = 5
	No = 1 Hardware: laptop = 4; desktop = 2; tablet = 3; mobile phone = 5	No = 0 Hardware: laptop = 3; desktop = 2; tablet = 3; mobile phone = 4
	Software: FaceTime = 6, Zoom = 1; Facebook video = 1	Software: FaceTime = 3; Zoom = 1; WhatsApp Video = 1
Cost of travel	£0.01–£10 = 6; £10.01–£20 = 1; £20.01–£40 = 1	£0 (free) = 2; £0.01–£10 = 2; More than £100.01 = 1
Preference score	9/9 F2F = 8	9/9 VC = 1; 8/9 VC = 1; 7/9 VC = 3

**Table 4:** Context for the consultation

Factor	*Participants accounts: Prefer F2F*	*Participants accounts: Prefer VC*
Pathway	*I’m not a particularly confident driver so I always go if I can avoid motorways and busy roads I do. Early in the morning that’s quite difficult because you’ve got all the people, well, you used to have all the people going to work and going to school. It would be the driving that would put me off an early morning one. I would rather leave home about nine when the traffic’s died down a bit* [3BV03] *But midday, physically it’s just because they have to keep in mind travelling and everything, that’s the only reason they want to have it midday, like one, two o’clock. Like I always wanted to have my appointment after two o’clock just because of the travelling.* [3BV04] *it (longer appointments) makes it more worthwhile. If you’re just perhaps going to be just checked up on what exercises you’re doing and then going, you’ve got to get there, park, get up the hill, which is a job in itself, and then wait around and then you’re only going to be five or 10 min and then you’re out again’* [3BF02] *Definitely I’ve had appointments where I felt that the clinician has been so thorough and made sure that they have done a thorough check and gone down every avenue to rule out things. You just feel that. You feel a bit better in yourself, because you feel that they’ve really been thorough … they give you time to ask questions or answer questions. It’s not rushed.* [3BF05] *You haven’t got quite so many obstacles with travelling if it’s in the middle of the day, it’s not so bad. But if you’re in the rush hour or you have a day at work you’re tired and just want to get home and so on.* [3BF02] *I would prefer when it’s physical, yeah, face-to-face because anything can happen within six months.* [3BF04] *I don’t know what the number is but there’s definitely a number around six or eight you don’t get more than. Dare I say if you’re using them up on phone appointments and then you end up with say four phone appointments and two physical appointments, that would just be silly and a waste of time* [3BF07]	*If I get the organised transport, the hospital transport, they require turning up four hours prior to that appointment even though it takes two hours. So sometimes I have been up at two/three in the morning ready for an early appointment, and then by the time I get there, having taken my morning medication, I am in a mess because it’s either not taken at the right time or … So yeah, that’s very difficult for me, and yes we have tried to change appointments to the middle of the day, sort of early afternoon if possible, and sometimes we’ve managed it.* [3BV06] *If I have appointment at 8:30 in the morning I have to leave my house at five o’clock or six o’clock in the morning and it’s a long journey for me. Because of the pain it would be really good if the physiotherapy team can call me and just carry on from that.* [3BV04] *Is it really worth it? Is it worth me going all that way to go for 15 min? I’m going to be seen for 15 min … it’s not going to be a thorough appointment. I won’t be seen. I won’t be checked properly. How is that possible for me to have 15-min appointment and then just go? You feel cheated, I think.* [3BF05] *It’s easy for me to get to and yes, it wouldn’t cost me that much by public transport. I can see why, if distance and cost was a major impact for a 15 min or half an hour appointment, then maybe you would prefer to have a virtual.* [3BF03] *People prefer to have first thing in the morning at home because then they have the whole day, or later in the evening when they can actually fit and have it either at the start of the day or the end of the evening, that’s why.* [3BV04]
Clinical	*So I think it’s worth having a face-to-face, but it’s also worth seeing someone who has the expertise, do you know what I mean?* [3BF07] *I’m in no pain. It isn’t like when I saw you that day. I was in bloody agony. So I needed a human being to physically see it and make it better. That’s what I think [unclear]. Do you know human beings rely on human beings as well? Unfortunately Zoom and COVID and everything will make us less human I’m afraid, potentially.* [3BF01] *Honestly, I’ve had my dodgy back since I was 15 and I’ve seen a lot of people. Honestly, I think for me, it’s too complicated to do over the phone, over the video. That just might be me, but I can also see other people with other problems where I’m actually thinking well, we probably could get away with that more so.* [3BF07] *But when I could barely walk the other week, it was never - I could never have done that - I would have felt that I was getting - I wouldn’t - it wouldn’t have felt right for me because he couldn’t have made me better virtually.* [3BF01] *I would just feel isolated. I would begin to feel isolated, and you become cut off from the outside world. You could just sit at home and have everything done at home and all your phone calls virtual, not face to face and everything. But where does that leave you with human interaction, social interaction? You’re just isolated.* [3BF05]	*Even if I’m in a car and I decide to drive, one day I got caught on the XX [motorway] coming to XX [hospital] and was stuck there for nearly two hours. Again, my pain levels were ridiculous for the rest of the week because it’s got a knock-on effect. It’s tough. That’s generally the thing that causes the problems.* [3BV01] *We’ve hit traffic accidents; we’ve hit loads of stuff on the way. So it is really difficult. Then really, i need a good hour of recovery before I see anyone when I gets there [laughs], to even being able to speak more coherently, if you know what I mean. Because pain takes over my speech and thinking process. So that’s really quite significant when you’re having a face-to-face; how much pain you are in to be able to communicate properly, if that makes sense* [3BV06] *Day-to-day activities is one of the areas that I do struggle with, but I’ve managed to find things to overcome it, and virtual is one of those things that help me to overcome the difficulties that I have with day-to-day living.* [3BV01] *I think I’d be more comfortable that way (having the camera off). I don’t like being viewed; I think … Yes, I don’t logically think I mustn’t look in a mirror. There’s always obviously a mirror in the bathroom but I’d never go and look at myself in a mirror, only when I’m washing my hands or whatever and the mirror’s there … Yes. When I was a bit younger I never ever wanted my photo taken.* [3BV02]

**Table 5:** Patient’s access to resources

Factor	Participants accounts: in favour of F2F	Participants accounts: in favour of VC
Socioeconomic factors	*No, I mean thanks to God I’m from a good family background so financially - yes, personally I mean obviously through the injury myself I’m [broke] down completely because I’m not working for three years but when it comes to travelling I think my family members they’ve been very supportive.* [3BV04] *I do think it link to their role at work. A lot of people with degrees are in occupations where it might be quite nice to have a paid morning off.. Also, we get paid. People who have got degrees tend to be in jobs where if you have half a day off or a day off for an appointment, (a) you’re covered by the Disability Act, the Equalities Act and (2) you get paid.* [3BV01] *For me, if I took the day off I will get paid. Where I work it would go down as a sick day and I would get paid. I’d be behind on my work, but I still would get my daily money for that, it wouldn’t cause me any hardship.* [3BF07] *So if I come in and see you at eight o’clock in the morning, I can come and see you, by nine o’clock I’ve left, by 10 o’clock I’m at work and my boss is quite happy.* [3BF07]	*Well, that would obviously be beneficial for them to have virtual, because they don’t know how many appointments they’re going to have. So if they’re having to go on a two-weekly basis for physio, they’re going to - I have to think twice before I go to an appointment. Before I got my disability badge, I had to think twice before I went to an appointment to Northwick Park Hospital, because the charges were so high for the car park.* [3BF05] *I’ve booked a cab four times - it cost me £200. One way is £125 … I would be like, oh no I can’t come because even dreams are impossible when you are injured, so the journey would be impossible for me because I wouldn’t be able to afford £100/£200 every week or every two weeks.* [3BF04] *I mean, if I’m coming from XX then obviously it’s quite far and it’s like more than 100 miles, I think, around 100 miles. Imagine if I were living further away, I would have to look for accommodation first.* [3BV04] *Whereas, if you’re in work like construction, for example, you don’t get paid if you’re self-employed, for having a day off. So, I think they’re the kinds of jobs that if you don’t get paid when you’re having a consultancy because you’re self-employed, you’re going to prefer a virtual. I think it links very much directly to employment roles, workload, and whether you get paid when you have the time off work for consultations.* [3BV01] *If I couldn’t get in my car and drive round the [x motorway], I can understand why it would be beneficial to be able to do it virtually.* [3BF07]
Equipment factors	*I’ve got my iPad set up and I don’t have a big room to set it up and a tripod and all that sort of stuff. So the video is always pointing slightly the wrong way and stuff like that, it’s not ideal. I think if we all did it more we’d be better kitted up for it.* [3BF07] *When you’re doing exercises and it’s running at about 10 frames a second or 20 frames a second, it’s just not very good … I don’t know whether it’s just because XX got a lot on the internet, because I’ve only used it once, but the frame rate and stuff is just shocking.* [3BF07] *Yes. I fall into that category, actually, because I’m not techy and whatever I seem to do with the phone goes wrong. Or the computer. Or the lighting system. I do have this strange effect on equipment* [3BF04] *I suppose if you take away the option of virtual, there’s only one option left for them. They just have one option. They haven’t got any other options. If they haven’t got the technology or haven’t got the equipment or don’t know how to work the technology, virtual is not going to work for them, is it?* [3BF01]	*But they’ve [older people] normally got a big and young family who teach them how to do it.* [3BV01] *They (older people) use it more than us at the moment [laughs]. If you look into it, like my dad, my mum, everybody, they are using Twitter, social media, Facebook and all that. I’m like, “oh my God they’re using it more than us”.* [3BV04] *Yeah, WhatsApp and all of those sorts of things. I do quite a lot of craft work and so we’ve been doing Zoom for that. I don’t think much work gets done, I think we just sit and yack* [3BF02] *I’ve been able to continue to work because I’ve been using the virtual meetings, which means that I can stay at home. So maybe that’s just already in my mindset, compared to other people who only have only known one thing and feel that if it’s - if they change that it might not be as good.* [3BV03] *I took the Zoom invite from my phone and then I just put the details on my work laptop. So I actually just typed in the meeting invite and the password and did it that way. That’s no problem, but I could have done it, I guess, from my phone. Just pressed - tapped on the link and then just gone straight through to that waiting room.* [3BV03]

**Table 6:** What’s required from the consultation

Factor	Participants accounts: in favour of F2F	Participants accounts: in favour of VC
Objective factors	*I still very much 100% think your first appointment should be a face-to-face. I would want the first one definitely so I would know what the exercises were (a) that you showed me what to do, but also that I was doing them correctly.* [3BF03] *I just don’t see how you can do it over the phone. Like I said, I think for follow-ups it’s not too bad, I don’t see how you could possibly do it only that, because I don’t see how you could ever assess someone for the first time without having a prod. But I’m not a physio, so I don’t know.* [3BF07] *But if it involves physical aspects where you’re having problems, has it changed, how is your knee looking or feeling now, can we see it move, then no, you have to go in for a check.* [3BF04] *Certainly, in a physio setting, somebody demonstrating how to do exercises or specific movements, I don’t know how the physio knows by virtual whether you’re actually doing them right or not.* [3BF03] *I would want a physical face-to-face appointment if I’m having a specific problem or a new issue. I mean, I’ve been there long enough that they know my condition and that’s fine.* [3BV03]	*In fact XX, a couple of weeks ago or last week, did actually phone out of the blue for an update. Everything that has been said previously they’ve sort of said again and it worked very well over the telephone* [3BV06] *It seems like I can do that over the internet much easier than I can in person. It feels like a waste of time, where this is quick. I can say what I need to, they can ask the questions, I can answer them, it takes 10/15 min out of my time, consultation over.* [3BV01] *But if I’m just coming up to be told a couple of things by someone looking on a screen and then saying this and that and then that’s it – then it is really pointless.* [3BV06] *At the moment the way my leg is I would be quite happy - if you were my physio today I would sit here and I would show you how far I can bend my knee backwards and what I can do with it. It’s not as swollen as it was* et cetera*,* et cetera*. I think that’s perfect* [3BF01] *I could teach anyone how to stretch their calves or their hamstrings, because I have to because I’ve got a dodgy back. That is less specialist and that is just the standard exercises out of the book, I’d call them.* [3BF04]
Interaction factors	*I still think it’s an age thing because I think it’s a security blanket going to see XX. You’ve built up that trust and that rapport over the last 20-odd years and you know they are doing their best for you.* [3BF01] *When you’re face-to-face, personally, I think you can engage better. You can see by people’s expressions, their movements, or their body language, which I don’t think you can always do when you’re on a virtual.* [3BF03] *Whether you’re gaining the therapist’s attention, full attention, as in - compared to a face-to-face. You can see what they’re doing. I do worry that there was other things going on. They were using the phone, answering other calls, or writing other texts, because you only see a head above.* [3BF03] *If I’ve had a telephone conversation, people can - it’s much easier to switch off what you’re saying or not hear clearly or misunderstand thing when you have a virtual one.* [3BF04] *I think that in itself is a kind of therapy, really, because when you - no matter what you’re going through, if someone else can see and are empathising with you, you start to feel a little bit better. You start to feel, well, someone here is concerned about me. They’re going to try their best to help me. I find the - I just find video calling a bit cold.* [3BF05] *I know things like Zoom has tightened up on their security. So maybe if they’re having more intimate type of examinations or having to remove clothing and all the rest of it, they might feel a bit uncomfortable doing that on a screen* [3BV03]	*I think the first appointment it’s always good to have virtually. The reason is that you can actually speak to your physio team and you can explain to them and they will be prepared, they will know that exactly. This patient, it’s their first appointment, you can speak to them virtually, they know exactly what the issues, what the problems are. They can have their own plan and let’s say the second appointment is face-to-face, so they know about you, they have a knowledge. Reading about you is one thing but speaking to you is another thing* [3BV04] *XX would know my knee was fine. There’s that trust, isn’t there. XX - [he’ll go, it’s alright XX] your knee is not bad at the moment. Next time we’ll just do it - over the iPhones or whatever. I’ll go yeah, yeah cool.* [3BF01] *If you’ve got a good rapport with them, and the patient gets confident that what they’re saying is true, then yeah, I think that [vc]'s a good option.* [3BF03] *Plus it can sort of read wrong results into it, where if you’ve travelled for a long time and you’re really hurting when you get there, then you’re not really showing the true average day as well, so I don’t know.* [3BV02] *They may have more success to have an interpreter within their own home and then they wouldn’t have to impinge on that person’s time, as well, to take them to the hospital with them* [3BF09]

**Table 7:** How COVID-19 influences preference

Factor	Participants accounts: in favour of F2F	Participants accounts: in favour of VC
Impact of COVID-19 on preference	*I’ve been into many hospitals, I’d never been to a hospital where it was so clean. I mean, the operation theatres, the wards - it was absolutely fantastic up there. You had complete confidence that you’re not going to get an infection, or you’re not going to come out with a problem. The nursing up there was fantastic* [3BF08] *Ninety per cent of the hospitals have got automatic doors, so you don’t have to touch anything. You go in, there’s somebody waiting for you in the reception area, they take you to see the person you want, and when that person’s finished with you, that person takes you back and lets you out through the front door* [3BF08] *If you said to me, can you come in? And I sit in the car, and you phone me and say, right, come in now, the door’s open, [unclear] walk straight into an office or wherever it is with you - I’m completely happy to do that.* [3BF08] *You just go in the safest environment you can get there in, whether it means that you go and, obviously, you wear a mask and you – I mean, the hospitals, themselves, I don’t think are any more riskier than going into Sainsbury’s or Tesco, so I can’t see, you know, they’ve got as much PPE as they’ve – obviously your – the people dealing with you are protected, and the environment themselves are cleaned as much as, and you’ve just got to be aware of what your surroundings are, haven’t you, really* [3BF09] *Once we have access to vaccinations, that’s it, back to normal. Everything. You’ll see the shopping centre, the hospitals packed, and people will forget about all these virtual appointments, I think.* [3BV04] *I think the NHS has always been about caring for the population and for people and everything, and when you don’t have - when you’re not going there physically and you’re not having that physically, it feels a bit cold. It feels a bit cold and just routine and not - I just feel that’s being lost from the country. I think that aspect of it is being lost. Everyone’s relying too much on technology, and we’re losing that whole human interaction* [3BF5]	*Before COVID I was discussing with my physiotherapy and occupational therapist that if she could provide any phone assistance or just, I mean video calls, because that would be easier for me because I was in a lot of pain throughout - it’s been three years since my injury. So, going there, coming back here it’s a long journey and so that’s why I was - when you guys approached me, I said, this is a really good thing to have. If it happens, really good.* [3BV04] *Everyone felt comfortable with it. But yeah Zoom is good. I know my wife has used it before - a long, long time before COVID. She was looking after some foreign buildings for a large corporate. There was obviously a lot of cost saving on jumping on airplanes backwards and forwards to different countries. Zoom costs £12 month.Flights cost a lot more. So yeah* [3BF01] *But because of COVID we are trying that now. That whole technology was sitting there but nobody was using it so because of COVID now everything has changed, like shopping, everything. Not only that, I mean if you look into it the technology is coming into like more than ever. Everyone is trying to get their - people who didn’t have smart phones, they’re getting smart phones, people who didn’t have laptops are getting laptops.* [3BV04] *Yeah. COVID has made us learn all new sorts of skills as well. We do a lot of client meetings through Zoom.* [3BF01] *I think because of the situation now with COVID it’s a completely different scenario from how it was before. So I suppose what my views were then and what my views are now are a bit different because obviously we don’t have that facility now that we did have* [3BF02] *In normal circumstances I would have driven in and I would have preferred to have driven in but because I haven’t been going out and feeling a bit nervous about going out, when you asked me, I thought no, I think I’d rather stay put. In another couple of weeks I’m sure I’ll be a lot more confident because I’m going to start going out a bit more* [3BV02] *From where I live which is, I don’t suppose you know XX, but well, this part of XX which is XX, to get up to London I have to go on a train and at least one tube, if not two tubes depending on where I’m going. No, I’m not prepared to do that... I’m not going on tubes.* [3BV02] *Look, unfortunately, we’re under very different circumstances at the moment. Yeah, it’s great to sit down with somebody across a table. … o this, this, and this, but on the phone what we’ve been doing is Zoom, sometimes your mind - you’re concentrating on something else, but it’s fine. I mean, I would like to sort of come up to Stanmore, sit down with you for the half an hour and run through everything, because you’ll have a [peg board] with you, but it’s not viable at the moment.* [3BF08] *At the moment I wouldn’t go to a hospital unless it was absolutely dire* [3BF02] *I would wait for COVID to finish because I’m not going to put myself in that position, or anybody else. Because you don’t know, it might not be you that becomes ill, but it might be someone else that you’ve effectively infected to make them very poorly* [3BF02] *Well I have said my reasons for wanting to visit a hospital. With this issue it’s completely new to us and I think you’ve just got to move with how it is to be sensible, to protect the hospital and its staff and its patients* [3BF02] *Yeah, and I think, as well, with the Coronavirus, I think a lot of things that people have had to go on to do virtually they would never have may be chosen to but have had no choice. They’ve done it and thought, well, you know* [3BF09]

### Context for the consultation

#### Pathway factors

Patients preferred virtual appointments early in the morning to avoid having to get up earlier and avoid rush hour traffic; public transport was busier during these times which was challenging for some patients and also led to patients preferring VC. Other patients however, preferred to get the appointment out of the way and were happy to travel. F2F appointments were easier later in the day as traffic volume would be reduced, there were fewer obstacles and there was a better chance of locating a parking space. Patients were less likely to prefer a F2F appointment for shorter durations, with some participants questioning whether it was ‘worth’ travelling in for only a 15-min appointment; longer appointments made travelling in more worthwhile. Some patients felt that they would rather a F2F appointment with a longer wait between sessions as ‘anything could happen’ during that space of time.

#### Clinical factors

Particularly for patients suffering from pain, avoidance of pain was a driver to prefer a VC. Patients who struggled with daily activities, especially getting ready in the morning, found travelling to an earlier appointment problematic. Extended travel led to an increase in pain which could last for several days and this led to some preferring VC.

Patients preferred to see a specialist F2F, particularly when symptoms were bad so that someone could physically assess them. There was a sense that VC was not suitable to address complex problems. The fear of being isolated is a motivating factor to attend consultations F2F. One participant expressed a general desire for a VC; their dislike of seeing themselves on a screen would lead them to opt for a phone rather than a video call.

*F2F* Face to face consultation, *VC* Virtual consultation, *F* Female, *M* Male

### Patient’s access to resources

#### Socioeconomic factors

The cost of travelling to the hospital is one reason for patients wanting to have a virtual consultation, particularly if repeated appointments are required. Travelling to an appointment was more costly for patients who did not have access to a car, particularly if they needed to travel on public transport during peak travel times, which tends to have a higher cost. Taxis were particularly costly for some patients and the requirement for overnight accommodation for a F2F appointment further influenced preferences in favour of a VC. A patient’s employment was a significant factor: some could afford to take time off work to attend appointments, while others would have to take unpaid leave. These financial factors influence preferences. Patients who had a degree were assumed to be paid higher than those who did not have a degree. Participants commented on how graduate jobs may have more chance of paid leave to attend appointments. More affluent patients were able to afford to take time out of work and attend a F2F consultation.

#### Equipment factors

Patients who lived with or near people who could support them with accessing or using equipment were in a stronger position to be able to use VC. Those patients who have been using technology for other areas of life and were familiar with it were more likely to choose VC than those who were not. Several patients reported an increased use of technology to communicate with work or family since the onset of the COVID-19 pandemic and would now consider using VC for their rehabilitation; particularly during the pandemic. Patients who did not have access to the equipment to conduct a VC were more likely to prefer a F2F consultation. In addition, poor internet connectivity was off-putting to patients.

### What’s required from the consultation

#### Objective factors

Respondents expressed they were happy to have a virtual consultation if a physical examination was not required. Participants were happy, in general, to have a VC for a discussion. It was recognized that a fluctuating condition might require different input at different times. Basic rehabilitation was acceptable to some, others preferred any form of rehabilitation to be carried out in person. First appointments were generally seen as better if they were conducted F2F, particularly if physical rehabilitation was required to ensure exercises were being completed correctly. Follow up appointments were deemed to be more acceptable via VC, particularly if the clinician was known to the patient. If an issue required a thorough assessment F2F was identified as the best option.

#### Interaction factors

One participant in the F2F group argued for first appointments to be conducted virtually to allow for a (subjective) assessment to be conducted to plan care. This was at odds with most of our DCE respondents who preferred F2F for their first appointment; this demonstrates the individual nature of preferences. Virtual care was best with a therapist who was known and trusted by the patient, with a good rapport facilitating preferences in favour of VC. Participants who reported travel to be a challenge described previous experiences where their interactions with healthcare professionals were inhibited by symptoms, such as the inability to focus on the content due to pain. It was thought that, for those patients who do not speak English, accessing a family member to support translation would be easier from home. Participants who had established relationships with their clinicians had confidence in VC. Interactions were better F2F rather than VC as it was easier to see body language. One participant referred to interactions as ‘cold’ virtually [3BF05] and several commented on how VC created the illusion of clinicians not listening as intently and potentially becoming distracted. Physically attending gave the potential for more empathy which was important. Intimate examinations over VC might make patients feel uncomfortable which may influence interactions.

### How COVID-19 influences preferences

VC during COVID-19 has provided patients with the opportunity to access their care virtually without the need for travel. For some, this was extremely positive. The pandemic highlighted the potential use of VC technologies and participants in this study thought that their use has increased across society. The potential benefits of VC in healthcare have become apparent to participants whereas these benefits were not previously visible. The healthcare and pandemic situation is different for the participants in this present study compared to when they completed the DCE (pre-pandemic). Due to this, participants stated they would answer the DCE differently if it were to be undertaken during the pandemic.

Participants were fearful of catching COVID-19 and could see that VC offers an opportunity to access care without being put under any undue risk of transmission. Travel, particularly on public transport, was seen as a high-risk activity for patients and some participants stated they would avoid this wherever they could. COVID-19 influenced patients’ preferences; many rationalised the trade-offs between travel and virtual care and although they would normally prefer F2F they would, under the current circumstances, opt for a VC. Despite this, a small number of participants expressed they would still travel in for their rehabilitation if this were available.

A hospital environment was viewed as a sterile, clean, place where there would be low risk of COVID-19 transmission. Participants cited infection control policies and procedures and would be happy to travel if they had access to their own transport. One participant suggested waiting in the car park until the clinician was ready to avoid spending unnecessary time within the hospital. Clinicians wearing Personal Protective Equipment inspired trust and one participant commented how they felt they were more likely to contract COVID-19 in a supermarket.

Although participants who were strongly in favour of F2F prior to the pandemic would consider undertaking a VC during COVID-19, they expressed a continued desire to have F2F consultations after the pandemic. Despite this, a greater appreciation of the potential benefits of VC was felt by all participants. It was felt by some participants that once the pandemic was over F2F care will become the norm once again.

## Discussion

Despite the DCE being terminated prematurely due to the COVID-19 pandemic, the results from the DCE suggested a tendency for certain patient groups to have preferences for VC [14]. A sub-sample group of participants with strong preferences for and against VC were identified from the DCE to participate in this present study. This study investigated the results of our previous DCE and provides additional useful insights. Thirteen participants (8 strongly in favour of F2F, 5 strongly in favour of VC) were interviewed to investigate the results of our DCE. In addition, several reasons why COVID-19 may have changed patients’ preferences towards VC during the pandemic were identified.

Our DCE [14] indicated that patients preferred VC when the therapist was known to the patient, there was a longer time until the next appointment, a shorter appointment early or late in the day; for patients without a degree, who had access to the equipment they need, had difficulty with day to day activities, were undergoing rehabilitation for multiple problem areas and hade to pay more than £5 for their return journey. Conversely, the opposite pre-conditions (when the therapist was not known to the patient, a shorter time until the next appointment, a longer appointment, in the middle of the day; for patients with a degree or above, did not have access to the equipment to undertake a virtual consultation, did not have difficulties with day-to-day activities, were undergoing rehabilitation for a single problem area and had less than £5 to pay for their return journey) led to patients preferring a F2F.

Patients preferred not to travel early in the morning for therapy if they had difficulty getting ready, had to wake up unacceptably early or did not like driving in rush hour. Ackerman and colleagues [17] identified that patients had preferences for certain times of day and this being a reason for not attending a self-management course. We have demonstrated how time of day can motivate preferences, with appointments in the middle of the day being easier for some due to reduced traffic and easier parking. Public transport can be more costly at peak times (i.e. early morning). Patients with musculoskeletal conditions may experience morning pain and stiffness [18]; these morning symptoms contributed towards patients preferring VC for an early appointment. Some patients may appreciate being able to spend time and energy gained from not travelling on other activities [19]. Elimination of transport time when using VC has been shown to be a significant benefit [15]. Our recent systematic review and qualitative synthesis [13] highlighted how changes in the work of being a patient influences preferences; if factors relating to travel and logistics make the work more burdensome for patients they are more likely to prefer an option that is less burdensome. PhysioDirect [20], a randomised trial investigating telephone advice and assessment services for physiotherapy, was more successful when calls were made at a convenient time for patients [21]. Time of appointment may not be a *true* reflection of preference for VC, rather the option of VC at that time making an appointment more convenient for patients at that time. Some patients in this study, however, liked an earlier appointment so they could travel in and get to work or other commitments earlier in the day. Some patients have reported the benefits of undergoing a Skype consultation from work [22]. Trends identified by our DCE do not apply to all, preferences are clearly individualised.

Being able to take paid time off work was important to allow F2F attendance with reduced financial burden. People in education to school leaving age are over represented in ‘zero hours’ contracts [23] and therefore may be unable to take paid leave for medical appointments. This may be challenging for some who have undergone surgery before their rehabilitation and been forced to take time off work previously [24]. A participant in our previous research [25] described how appointments had become a full time job; repeated attendance can get in the way of employment and travel can be financially demanding.

Equipment can be costly; ‘Attend Anywhere’, the platform of choice across the NHS in England and Scotland, requires Windows 7+, MacOS 10.11+ (released 2009 and 2015 on Windows and Mac respectively) on a desktop and Android 5.1+, iOS 12.4+ (released 2015 and 2019 on Android and Apple phones or tablets respectively) with up to date Chrome or Safari software [26]. A patient’s financial position may remove the opportunity for VC through the initial purchase and the ongoing costs of some software that drive up data usage costs. When outdated hardware was incompatible with the platform, this led to reduced patient satisfaction [2].

Many of the patients in this study preferred to have a F2F prior to a VC, although one patient reported they would be happy for an initial assessment. Other studies [15, 22] reveal how patients favoured initial F2F appointments prior to VC. For our patients, this was to conduct a thorough assessment and to learn the correct exercises. If a patient was experiencing a worsening of symptoms, they are more likely to want a F2F. VC offers flexibility [27] but patients might also want a F2F to identify the cause of a new problem should it arise [22]. Some patients felt that VC would not be accurate whereas Cottrell and colleagues [28] found high levels of agreement between in-person assessment of patients and telehealth appointments. Teleconference goniometry has been shown to be as accurate as in person goniometry of the elbow [29].

Therapists are forced to rely on their talking and listening skills (as opposed to hands on) which may be problematic for patients with communication difficulties; ordinary conversation has been demonstrated to be a key factor of a therapeutic relationship [30]. One participant in this study reported not liking seeing themselves on a screen, participants with social anxiety disorder have been shown to have self-focused attention during conversations using Skype [31]. Patients have been shown to be skeptical about telephone appointments prior to use [21, 32]. However, trying out a VC platform has been shown to increase positivity about ease of use and usefulness compared to those who did not use it [15] and may alter the perception of the patient-therapist relationship [15].

Nationally in orthopaedics, routine care was abandoned due to COVID-19 to reduce patient ‘flow’ to prevent the NHS being overwhelmed [33]. The NHS now faces an estimation of 400,000 procedures not being performed every month [34]. Virtual orthopaedic consultations have subsequently been hastened and rapidly implemented [2], with new guidance for virtual care being disseminated widely to support use in orthopaedics [35]. Patients in this study indicated that their stated preferences in our Discrete Choice Experiment (conducted between December 2019 and March 2020) would have been more favourable towards VC if they were able to foresee the impact of the pandemic. Patients did not feel F2F was viable during the height of the pandemic and were not happy to take public transport. Patients were using platforms like Zoom and WhatsApp to communicate with friends and family and reported they felt more confident with using VC to access care; prior to the pandemic some patients had not used these technologies. Using VC highlighted the benefits of not travelling and saving money. Some patients, however, would still be happy to travel for a F2F appointment as they believed the risks of transmission would be low with proper precautions. It was suggested by some that everything would return to normal after the pandemic subsided and F2F would resume once more. Interestingly, of those patients who were unable to have a F2F due to COVID-19, less than half of VC patients would prefer a VC next time [2]. The pandemic has affected preferences in the short term, what is not clear is how preferences will be affected in the long term.

### Strengths and limitations of this study

Our previous DCE results indicated factors that influenced preference for VC or F2F. The interview schedule and coding frame from this present study reflected this, and it is possible that different or additional questions may have yielded different results. Of the participants eligible for inclusion, 68% did not respond to the initial or follow-up email; an increased number of participants may have changed our conclusions. The limited pool of participants who had strong preferences for VC reduced our potential sample and as a result the recruitment reflects a larger number of participants in favour of F2F from our DCE. An alternative sampling strategy may have led to a higher level of recruitment than was observed in this study. Higher recruitment numbers may have influenced our conclusions. Despite these limitations, we have been able to sample groups of patients who were able to offer a diverse range of perspectives. We have used theoretically informed qualitative methods to interpret a DCE through interviewing these participants to understand what they think these results mean. These results will be of particular interest to the physiotherapy and rehabilitation community who are using virtually supported consultations in their patient pathways.

## Conclusion

This paper presents a study that investigated the results of a discrete choice experiment and has explored the impact of COVID-19 on patient preferences for VC. Patients suggested a range of potential reasons as to how the context of the consultation, patient’s access to resources and the requirements of the consultation might impact their preference. In addition, patients shared experience and viewpoints on how the COVID-19 pandemic has influenced preferences for VC. VC during COVID-19 has provided patients with the opportunity to access their care virtually without the need for travel. For some, this was extremely positive as it provided opportunities to access care without the need for F2F social interactions and potentially risk contracting the virus. Many felt that VC would become more commonplace after the pandemic whereas others were keen to return to F2F consultations as much as possible. This research sheds light on some of the underlying rationale behind patient preferences for VC in certain situations.

## Supplementary Information

**Additional file 1.** .

## Data Availability

The datasets used and/or analysed during the current study are available from the corresponding author on reasonable request.

## Abbreviations

DCE: Discrete Choice Experiment
F2F: Face-to-face consultation
VC: Virtual consultation
