# Supplementary material for: A qualitative investigation into the results of a discrete choice experiment and the impact of COVID-19 on patient preferences for virtual consultations
Source: Arch Physiother. 2021 Sep 6;11:20. doi: 10.1186/s40945-021-00115-0 (PMC8419808; doi:10.1186/s40945-021-00115-0)
Supplement: Supplementary file 1 — Additional file 1. . [file 40945_2021_115_MOESM1_ESM.docx]

**CONNECT Phase 3 Project Topic Guide**

**Part 1 – Results of Phase 3**

- Individual responses
- (discuss with participant)
- Impact of COVID on individual responses

**Part 2 – Results of Phase 3**

- Group responses
- (discuss with participant)
- Impact of COVID on group responses

**Part 3 – Burden of Treatment**

- Impact on Patient: how technology consultations influence the experience of living with illness and engagement with clinical care

**Part 4 – Virtual Clinic Design**

- Design considerations: what should virtual consultation pathways look like in practice?
